# Supplementary material for: Application of High-Throughput Sequencing Technology in the Pathogen Identification of Diverse Infectious Diseases in Nephrology Departments
Source: Diagnostics (Basel). 2022 Sep 1;12(9):2128. doi: 10.3390/diagnostics12092128 (PMC9497786; doi:10.3390/diagnostics12092128)
Supplement: Supplementary file 1 [file diagnostics-12-02128-s001.zip › diagnostics-1814342-supplementary.pdf]

Supplemental Table S1. Identified pathogens in patients with UTI, PDAP, CRBI and lung infection by HTS and culture methods.

Pathogens \ Diseases	UTI, n (%)		PDAP, n (%)		CRBI, n (%)		Lung infection, n (%)	
	HTS	Culture	HTS	Culture	HTS	Culture	HTS	Culture
Gram-positive bacteria								
Enterococcus faecium	12 (5.69)	3 (6.98)	2 (2.94)	1 (6.25)	-	-	3 (2.70)	-
Enterococcus faecalis	11 (5.21)	2 (4.65)	-	-	-	-	2 (1.80)	-
Finegoldia magna	9 (4.27)	-	1 (1.47)	-	-	-	-	-
Streptococcus	6 (2.84)	1 (2.33)	7 (10.29)	-	-	-	22 (19.82)	-
Lactobacillus inert	6 (2.84)	-	2 (2.94)	1 (6.25)	-	-	4 (3.60)	-
Peptoniphilus harei	3 (1.42)	-	-	-	-	-	-	-
Corynebacterium	3 (1.42)	-	4 (5.88)	-	3 (13.64)	-	3 (2.70)	-
Corynebacterium pyruvate	3 (1.42)	-	-	-	-	-	-	-
Bifidobacterium	2 (0.95)	-	-	-	-	-	-	-
Staphylococcus haemolyticus	2 (0.95)	-	-	-	-	-	-	-
Lactobacillus	2 (0.95)	-	-	-	-	-	-	-
Micromonas minimus	1 (0.47)	-	-	-	-	-	-	-
Enterococcus gallinarum	1 (0.47)	1 (2.33)	-	-	-	-	-	-
Staphylococcus epidermidis	1 (0.47)	-	4 (5.88)	-	-	-	-	-
Staphylococcus warneri	1 (0.47)	-	-	-	-	-	1 (0.90)	-
Enterococcus avium	1 (0.47)	-	-	-	-	-	-	-
Staphylococcus aureus	-	-	3 (4.41)	4 (25.00)	5 (22.73)	5 (71.43)	4 (3.60)	1 (33.3)
Peptophilus	-	-	2 (2.94)	-	-	-	-	-
Abiotrophia defectiva			1 (1.47)	-	-	-	4 (3.60)	-
Aerococcus viridans	-	-	1 (1.47)	-	-	-	-	-
Bacillus cereus	-	-	1 (1.47)	-	-	-	1 (0.90)	-
Staphylococcus capitis	-	-	1 (1.47)	-	-	-	-	-
Anaerococcus	-	-	-	-	2 (9.09)	-	-	-

Glycolytic Staphylococcus	-	-	-	-	1 (4.55)	-	-	-
Bacillus thuringiensis	-	-	-	-	1 (4.55)	-	-	-
Streptococcus pneumoniae	-	-	-	-	1 (4.55)	-	-	-
Streptococcus sanguis	-	-	-	-	1 (4.55)	-	-	-
Microbacterium paraoxydans	-	-	-	-	-	1 (14.29)	-	-
Stomatococcus mucilaginosus	-	-	-	-	-	-	7 (6.31)	-
Granulicatella adiacens	-	-	-	-	-	-	2 (1.80)	-
Leuconostoc Leuconostoc	-	-	-	-	-	-	2 (1.80)	-
Streptococcus granulosus	-	-	-	-	-	-	2 (1.80)	-
Staphylococcus glycolyticus	-	-	-	-	-	-	1 (0.90)	-
Gemella haemolysans	-	-	-	-	-	-	1 (0.90)	-
Gordonia	-	-	-	-	-	-	1 (0.90)	-
Lactobacillus Rossi	-	-	-	-	-	-	1 (0.90)	-
Gram-negative bacteria								
Escherichia coli	36 (17.06)	19 (44.19)	5 (7.35)	3 (18.75)	-	-	2 (1.80)	1 (33.3)
Gardnerella vaginalis	12 (5.69)	-	-	-	-	-	-	-
Campylobacter urealyticum	6 (2.84)	-	-	-	-	-	-	-
Klebsiella pneumoniae	9 (4.27)	3 (6.98)	2 (2.94)	-	-	-	-	-
¶ *Prevotella*	7 (3.32)	-	-	-	-	-	-	-
Atopobium vaginalis	5 (2.37)	-	-	-	-	-	-	-
Proteus mirabilis	5 (2.37)	-	-	-	-	-	-	-
Haemophilus parainfluenzae	2 (0.95)	-	-	-	-	-	2 (1.80)	-
Bacteroides	2 (0.95)	-	-	-	-	-	-	-
Aeromonas caviae	2 (0.95)	-	-	-	-	-	-	-
Morganella morganii	3 (1.42)	1 (2.33)	1 (1.47)	-	-	-	-	-
Acinetobacter baumannii	2 (0.95)	1 (2.33)	-	-	-	-	2 (1.80)	-
Stenotrophomonas maltophilia	3 (1.42)	-	1 (1.47)	1 (6.25)	-	-	-	-
Enterobacter cloacae	1 (0.47)	-	-	-	-	-	-	-

Neisseria	1 (0.47)	-	2 (2.94)	-	-	-	5 (4.50)	-
Veillonella parvula	1 (0.47)	-	-	-	-	-	2 (1.80)	-
Pseudomonas aeruginosa	1 (0.47)	1 (2.33)	4 (5.88)	3 (18.75)	-	-	1 (0.90)	-
Chryseobacterium indolegenes	1 (0.47)	-	-	-	-	-	-	-
Alcaligenes faecalis	1 (0.47)	-	-	-	-	-	-	-
Serratia marcescens	1 (0.47)	1 (2.33)	-	-	-	-	-	-
Citrobacter freundii	1 (0.47)	1 (2.33)	-	-	-	-	-	-
Rhodococcus sphaeroides	-	-	1 (1.47)	-	-	-	-	-
Pseudomonas nitrogens	-	-	1 (1.47)	-	-	-	-	-
Acinetobacter lwoffi	-	-	1 (1.47)	-	-	-	2 (1.80)	-
Moraxella *osloensis*	-	-	1 (1.47)	-	-	-	-	-
Trichomonas spp	-	-	1 (1.47)	-	-	-	-	-
Pseudomonas oryzihabitans	-	-	1 (1.47)	-	-	-	-	-
Pseudomonas umsongensis	-	-	1 (1.47)	-	-	-	-	-
Paracoccus marinus	-	-	1 (1.47)	-	-	-	-	-
Rhizobium radiobacter	-	-	-	-	1 (4.55)	1 (14.29)	-	-
Acinetobacter variabilis	-	-	-	-	1 (4.55)	-	-	-
Paracoccus	-	-	-	-	1 (4.55)	-	-	-
Hafnia alvei	-	-	-	-	1 (4.55)	-	-	-
Burkholderia cepacian	-	-	-	-	1 (4.55)	-	-	-
Citrobacter	-	-	-	-	-	-	2 (1.80)	-
Haemophilus influenzae	-	-	-	-	-	-	1 (0.90)	-
Veillonella atypica	-	-	-	-	-	-	1 (0.90)	-
Prevotella jejuni	-	-	-	-	-	-	1 (0.90)	-
Paracoccus evansi	-	-	-	-	-	-	1 (0.90)	-
Rhizobia	-	-	-	-	-	-	1 (0.90)	1 (33.4)
Fungus								

Candida albicans	6 (2.84)	3 (6.98)	-	-	-	-	7 (6.31)	-
Candida parapsilosis	4 (1.90)	3 (6.98)	3 (4.41)	1 (6.25)	1 (4.55)	-	4 (3.60)	-
Trichosporon asahii	4 (1.90)	-	-	-	-	-	-	-
Pichia	3 (1.42)	-					1 (0.90)	-
Candida glabrata	3 (1.42)	1 (2.33)	-	-	-	-	-	-
Aspergillus	2 (0.95)	-	5 (7.35)	1 (6.25)	-	-	3 (2.70)	-
Candida tropicalis	1 (0.47)	1 (2.33)			1 (4.55)	-	1 (0.90)	-
Candida rugosa	1 (0.47)	-	-	-	-	-	-	-
Candida krusei	-	1 (2.33)	-	-	-	-	-	-
Aureobasidium	-	-	1 (1.47)	-	-	-	-	-
Candida sake	-	-	1 (1.47)	-	1 (4.55)	-	-	-
Yarrowia lipolytica	-	-	1 (1.47)	-	-	-	2 (1.80)	-
Malassezia	-	-	1 (1.47)	-	-	-	-	-
Exophiala dermatitidis	-	-	1 (1.47)	1 (6.25)	-	-	-	-
Clavispora lusitaniae	-	-	-	-	-	-	1 (0.90)	-
Cyanobacteria cordiformis	-	-	-	-	-	-	1 (0.90)	-
Trichospora epidermidis	-	-	-	-	-	-	1 (0.90)	-
Saccharomyces cerevisiae	-	-	-	-	-	-	1 (0.90)	-
Ochroconis	-	-	-	-	-	-	1 (0.90)	-
Pneumocystis jiroveci	-	-	-	-	-	-	1 (0.90)	-
Other								
Ureaplasma urealyticum	4 (1.90)	-	-	-	-	-	-	-
Ureaplasma parvum	4 (1.90)	-	-	-	-	-	-	-
Mycoplasma hominis	3 (1.42)	-	-	-	-	-	-	-
Mycobacterium tuberculosis	-	-	2 (2.94)	-	-	-	-	-
Rickettsia bellii	-	-	1 (1.47)	-	-	-	-	-
Oral Mycoplasma	-	-	-	-	-	-	1 (0.90)	-
Cryptosporidium	-	-	-	-	-	-	1 (0.90)	-

Mycobacterium Gordon	-	-	-	-	-	-	1 (0.90)	-
Total	211 (100)	43 (100)	68 (100)	16 (100)	22 (100)	7 (100)	111 (100)	3 (100)

n: number of pathogen isolates.

Supplemental Figure S1

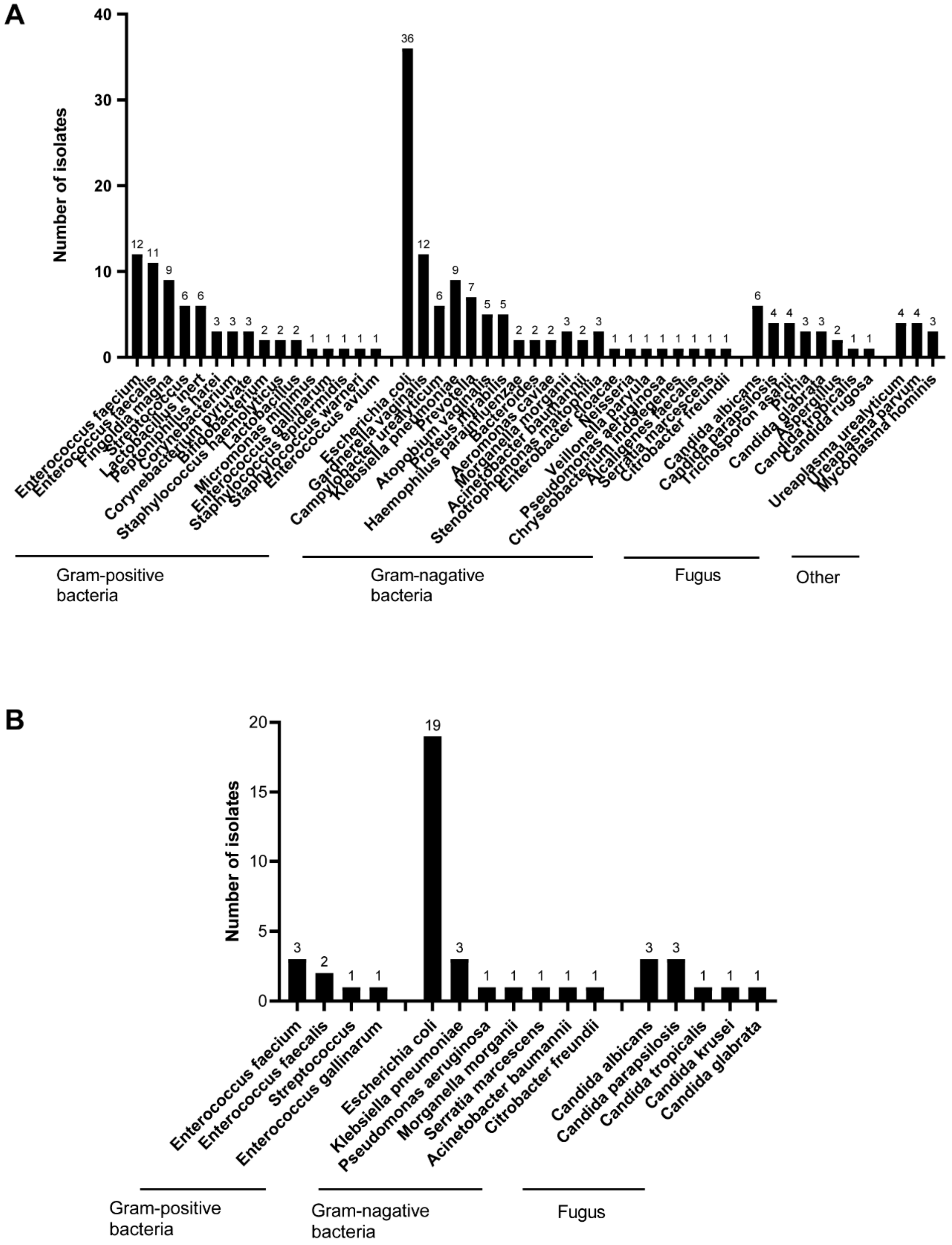

Supplemental Figure S1. Profile of identified pathogens in patients with UTI by method of HTS (A) and culture method (B).

Supplemental Figure S2

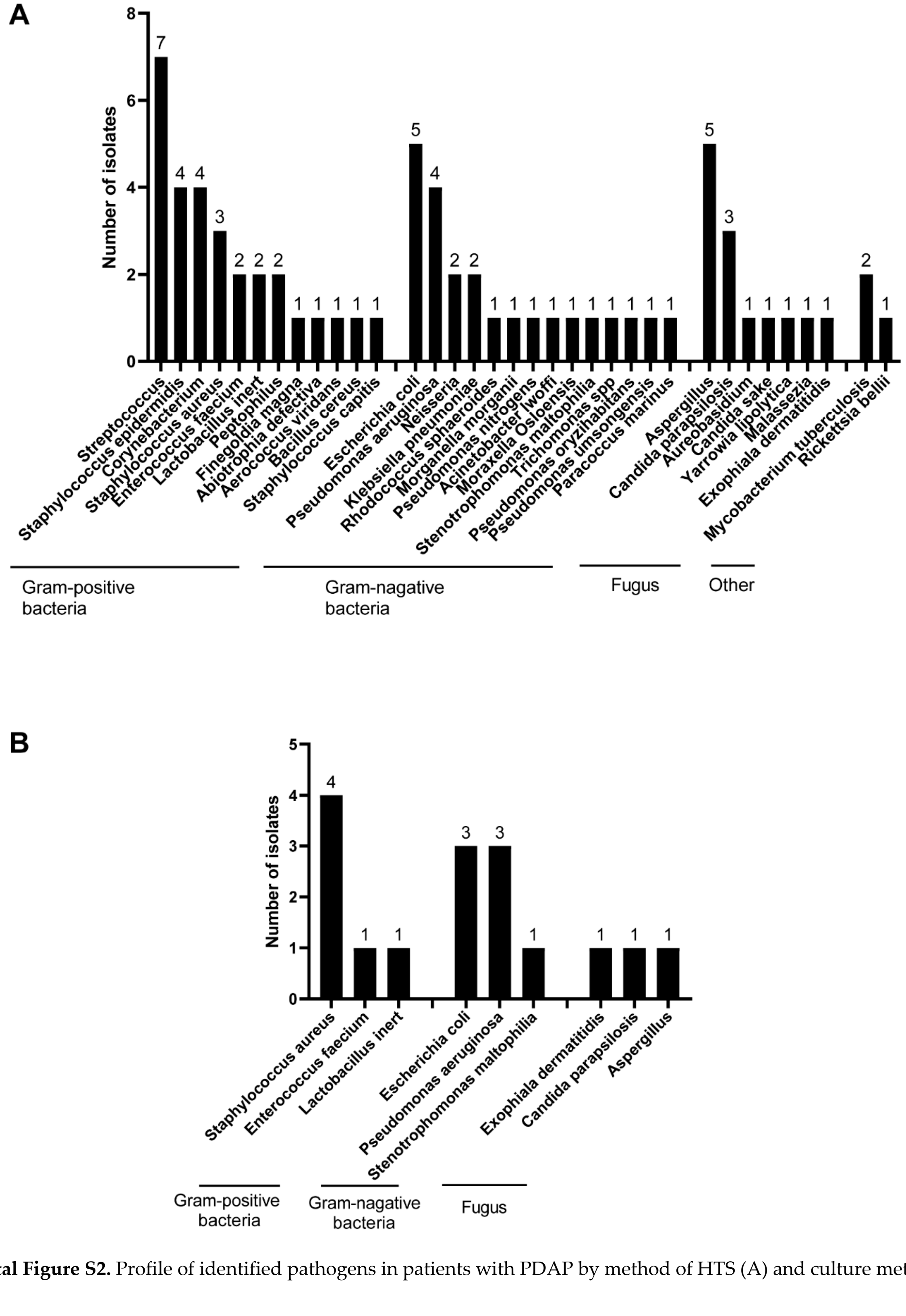

Supplemental Figure S2. Profile of identified pathogens in patients with PDAP by method of HTS (A) and culture method (B).

Supplemental Figure S3

A

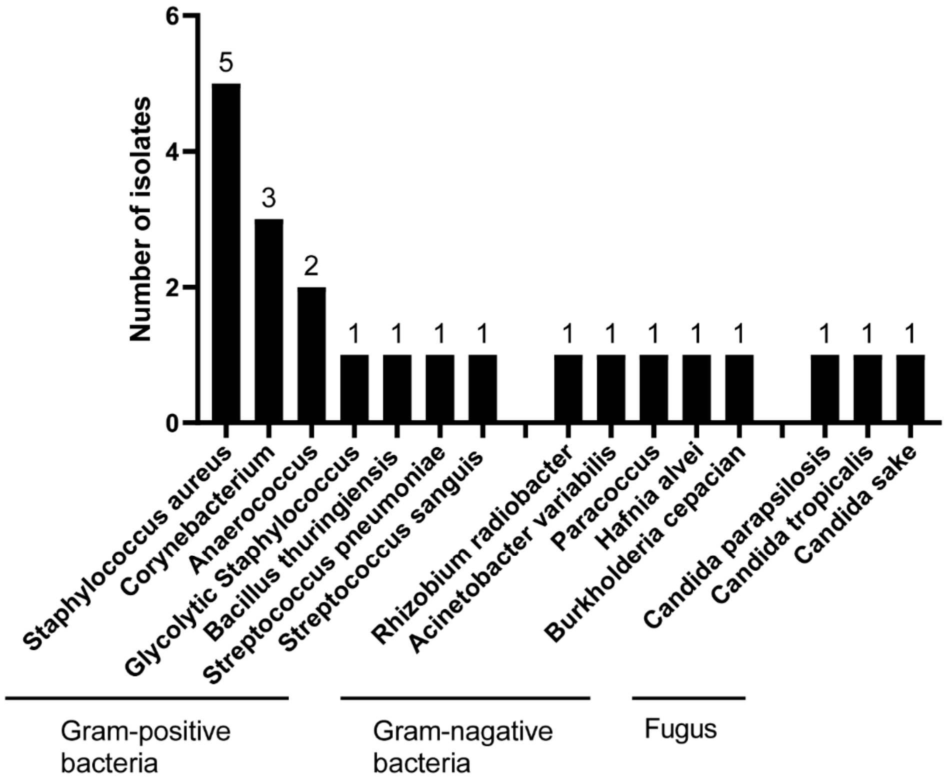

B

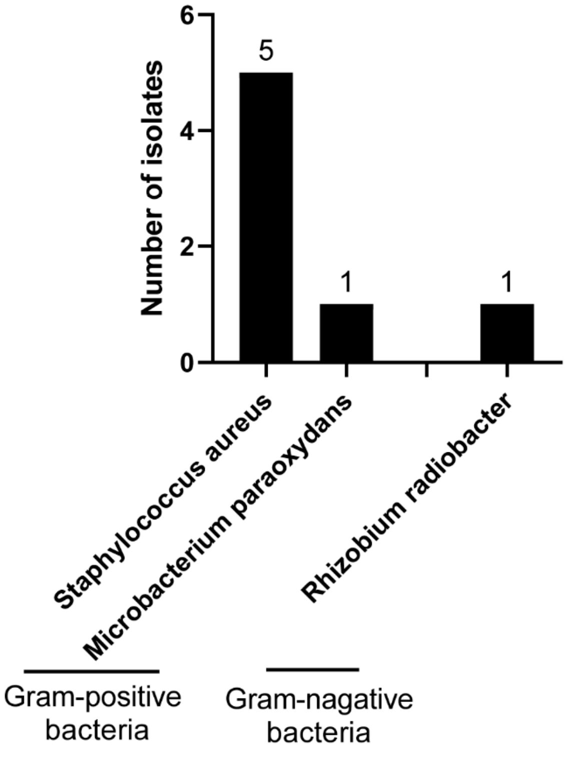

Supplemental Figure S3. Profile of identified pathogens in patients with CRBI by method of HTS (A) and culture method (B).

Supplemental Figure S4

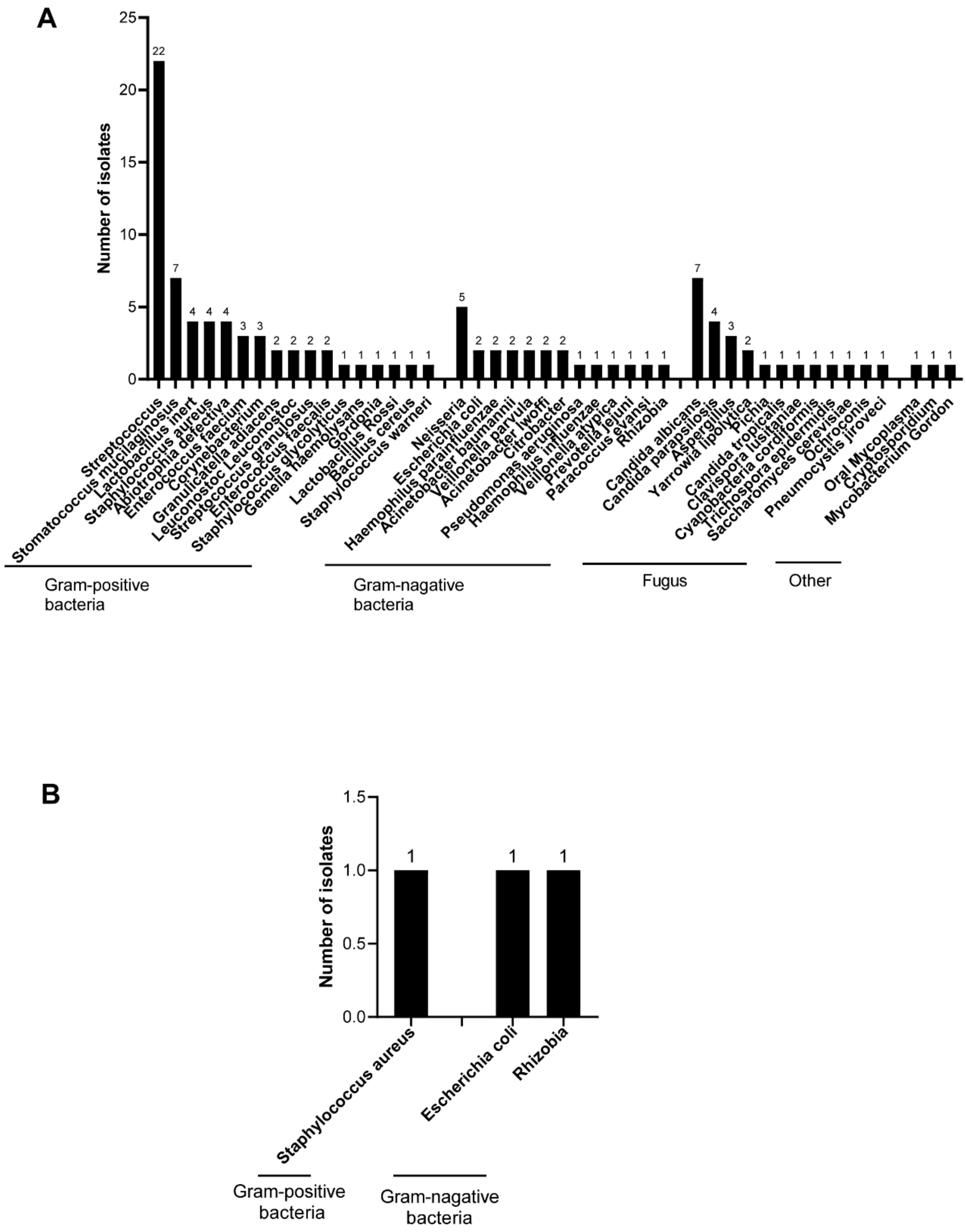

Supplemental Figure S4. Profile of identified pathogens in patients with lung infection by method of HTS (A) and culture method (B).
